# Impact of socioeconomic deprivation on risk and disease activity of Sjögren’s disease

**DOI:** 10.1136/rmdopen-2025-006348

**Published:** 2026-03-04

**Authors:** Aliaksandra Baranskaya, Matilde Bandeira, Abdullah Nadeem, Simon J Bowman, Valentina Pucino, Benjamin A Fisher

**Affiliations:** 1 Rheumatology Research Group, School of Infection, Inflammation and Immunology, University of Birmingham, Birmingham, UK; 2 NIHR Birmingham Biomedical Research Centre, University Hospitals, Birmingham, UK; 3 Department of Rheumatology, Queen Elizabeth Hospital, University Hospitals Birmingham NHS Foundation Trust, Birmingham, UK; 4 Rheumatology Department, Unidade Local de Saúde Santa Maria, Centro Académico de Medicina de Lisboa (CAML), Lisboa, Lisboa, Portugal; 5 Faculdade de Medicina, Universidade de Lisboa, CAML, Lisboa, Portugal; 6 University of Leicester Medical School, University of Leicester, Leicester, UK; 7 Immunoallergology Unit, Department of Clinical and Experimental Medicine, University of Pisa, Pisa, Italy

**Keywords:** Epidemiology, Sjogren's Syndrome, Social work, Public Health

## Abstract

**Background:**

We aimed to assess the impact of socioeconomic status (SES) on risk of Sjögren’s disease (SjD) compared with non-Sjögren’s Sicca and population controls, and on the clinical features of SjD.

**Methods:**

A single-centre UK cohort provided participants with SjD (European Alliance of Associations for Rheumatology/American College of Rheumatology (EULAR/ACR) 2016, n=256) and non-Sjögren’s Sicca (anti-Sjögren’s syndrome type A (SSA)/Ro negative, n=175). Health Survey for England 2019 provided local population controls (n=972). English Indices of Multiple Deprivation (IMD) 2019 quintiles at recruitment and highest educational attainment defined SES.

Adjusted logistic regression models evaluated associations between SES and diagnosis. Linear models assessed the impact of IMD on disease variables. Population controls were matched with age, sex and ethnicity to compare SES distributions.

**Results:**

Across IMD and educational attainment, participants with SjD had lower SES status compared to Sicca (p=0.008 and p=0.018). Odds of SjD (vs Sicca) were highest in most deprived IMD quintile 1 and reduced by 74% in quintile 2 (OR 0.26 (0.12, 0.58), p<0.001).

Immunoglobulin G and A levels were inversely associated with IMD. In SjD, each unit increase in IMD reduced IgG by 6.03% (−9.84%, −2.05%; p=0.003) and IgA by 6.46% (−10.87%, −1.60%; p=0.010).

When compared with population controls, IMD was not a risk factor for SjD (p=0.257) whereas Sicca was associated with lower deprivation (p=0.003). Those with a degree level qualification had the highest odds of diagnosis (SjD or Sicca).

**Conclusions:**

Low SES is associated with increased risk of SjD compared to Sicca and with higher immunoglobulin levels. The Sicca cohort may be less deprived than the general population. The role of environmental factors in modulating salivary gland pathology requires further exploration.

**WHAT IS ALREADY KNOWN ON THIS TOPIC**

- Socioeconomic deprivation has been associated with an increased risk of developing some rheumatic diseases and with higher disease activity. However, focused literature on effects of socioeconomic deprivation on Sjögren’s disease (SjD) is lacking.

**WHAT THIS STUDY ADDS**

- Higher levels of socioeconomic deprivation are associated with increased risk of SjD compared with non-SjD Sicca and biologically, with higher immunoglobulin levels, which are markers of disease activity. Comparison with population controls found non-SjD Sicca to be associated with lower socioeconomic deprivation defined by IMD.

**HOW THIS STUDY MIGHT AFFECT RESEARCH, PRACTICE OR POLICY**

- Our work introduces a hypothesis that exposures arising from socioeconomic deprivation may influence the clinical phenotype of salivary gland pathology. It also highlights the importance of control population selection and routine reporting of socioeconomic data in SjD research.

## Introduction

 Sjögren’s disease (SjD) is a multisystem autoimmune disease characterised by lymphocytic infiltration of exocrine glands. It presents primarily with dryness but may be accompanied by fatigue, major organ complications and lymphoma,^1^ alongside reduction in quality of life and significant economic burden.^1 2^ The aetiology of SjD is poorly understood. Although environmental risk factors are thought to substantively contribute to risk, these are ill-defined with possible roles for infection (such as Epstein Barr Virus), hormones, diet, negative stressful life events, tonsillectomy, air pollutants and negative associations with smoking when compared with disease controls.^3^,^10^

Evidence is emerging that socioeconomic status (SES), in particular socioeconomic disadvantage or deprivation, is a risk factor for many immune and non-immune-mediated diseases.^11 12^ SES is a multidimensional concept that represents an individual’s position within a society, which may influence their access to resources, exposures to environmental hazards and more. SES can be defined by various surrogate measures at both the individual and community/area level, as well as at different timepoints of an individual’s lifespan (childhood vs adulthood). Commonly used individual indicators are education, occupation and income, which can also be used at the parental/household level.^12^,^15^ Examples of area-based SES measures include English Index of Multiple Deprivation (IMD), Townsend Deprivation Index and, more broadly, the country’s Gross Domestic Product (GDP) per capita.

Socioeconomic deprivation has been associated with an increased risk for rheumatoid arthritis, a commonly studied rheumatic disease, as well as higher disease activity^12 14 15^ and worse long-term functional outcomes.^16^ However, focused literature on SES and SjD is lacking.

We aimed to use a well-characterised SjD cohort to assess for associations between socioeconomic status and the following:

1. Risk of SjD (compared with non-SjD Sicca and general population).
2. Clinical features of SjD.

## Methodology

### Cohorts

This study was undertaken using the Optimising Assessment in Sjögren’s Syndrome (OASIS) cohort. Since 2014, OASIS has recruited patients presenting with sicca symptoms or other features warranting investigation for SjD to a multidisciplinary SjD clinic at the Queen Elizabeth Hospital Birmingham, Birmingham, United Kingdom. At enrolment into OASIS, demographic data, symptom duration and disease activity scores, including the European Alliance of Associations for Rheumatology (EULAR) SjD Disease activity Index (ESSDAI), are collected. Tear production is assessed by Schirmer’s test without anaesthetic, and unstimulated salivary flow over 5 min is measured. As part of the initial assessment, subjects are given questionnaires that include patient reported outcomes, including the EULAR SjD Patient Reported Index (ESSPRI) and questions on hypothetical risk factors. Data on focus score (number of lymphocytic foci per 4 mm^2^ tissue)^17^ are collected from those participants who undergo a minor salivary gland biopsy for diagnostic purposes.

For this study, SjD was defined by fulfilment of EULAR/ACR 2016 classification criteria^18^ for SjD and having a physician diagnosis of SjD in the absence of other systemic connective tissue disease. Non-SjD Sicca patients (Sicca) did not have a physician diagnosis of SjD or fulfil classification criteria and were anti-Ro/SSA antibody negative. Patients with exposure to immune checkpoint inhibitors were excluded.

Health Survey for England (HSE) 2019 data^19^ were used to derive population controls based in West Midlands. HSE is a governmentally run annual survey of the English population that monitors health behaviour trends. Data are collected via an interview and reported with geographical granularity to region. The survey defines individuals aged ≥16 as adults. The variables used from the HSE survey are described in online supplemental methods.

### Socioeconomic variables

SES of the participants was defined by two separate measures: English Index of Multiple Deprivation (IMD) 2019 and highest educational attainment. Two SES markers were used as agreement between individual and area-based SES measures has previously been found to vary.^11 20^ We used 2019 HSE cohort data as 2018 was calculated as the median recruitment year for the OASIS cohort, and the IMD was updated in 2019 allowing comparability between population and OASIS data.

IMD is a postcode-based composite measure of relative deprivation for a small geographical area of ~1500 people in England.^21^ It is used by the Ministry of Housing, Communities and Local Government to assist in policy making and monitor population deprivation. All areas in England are ranked based on their level of deprivation and these ranks are used to produce deciles. These can be reported as quintiles whereby quintile 1 represents 20% most deprived areas nationally and quintile 5 represents 20% least deprived areas nationally. The IMD index is composed of seven deprivation domains with two supplementary income-based domains. Each domain offers a differing weight contribution to overall score and can be independently reported as deciles/quintiles:

- Income (22.5% appropriate weight contribution) - plus supplementary Income Deprivation Affecting Children Index (IDOACI) and Income Deprivation Affecting Older People Index (IDOPCI).
- Employment (22.5%).
- Education, skills and training (13.5%).
- Health deprivation and disability (13.5%).
- Crime (9.3%).
- Barriers to housing and services (9.3%).
- Living environment (9.3%).

Postcodes at time of recruitment into OASIS were used to generate IMD quintiles using the open access 2019 IMD postcode lookup tool.^22^ HSE dataset reports IMD quintiles for all participants at time of recruitment.

Highest educational attainment was self-reported in a baseline OASIS questionnaire and in HSE survey. In this study, it is categorised as no lower education qualification, lower secondary qualifications (ie, GCSEs/O levels, or equivalent to Level 1 or 2 UK qualification levels)^23^, upper secondary qualifications (ie, A levels or equivalent to Levels 3–5) and degree or above (Levels 6–8)).

### Disease variables

The following disease variables were assessed for SjD and Sicca cohorts: immunoglobulin levels (IgA, IgG and IgM), rheumatoid factor (RF), focus score, unstimulated salivary flow rate (ml/min), mean Schirmer test, ESSPRI and Hospital Anxiety and Depression Scale (HADS) scores for anxiety and depression separately. Mean Schirmer was calculated as the average of the two eye scores for each patient, using the single eye score if only one value was available. The ESSDAI total score was analysed for the SjD group only. Although a focus score is typically only calculated in the presence of a histological pattern of lymphocytic sialadenitis,^17^ for the purpose of this study we calculated focus scores for the Sicca population also, including in the numerator all foci >50 cells even when the predominant tissue pattern was one of atrophy and fibrosis.

### Statistical analysis

Categorical variables have been reported as frequencies and percentages, and continuous variables have been reported as medians with IQRs or means with SD. All statistical analyses were undertaken using IBM SPSS statistics V. 29.0.0.0. P values ≤0.05 were deemed statistically significant for all tests.

The Jonckheere-Terpstra test was used to assess for differences across IMD quintiles for each disease variable (such as ESSDAI).

#### Assessment of disease risk

Binary logistic regression models were used to investigate the risk of SjD vs Sicca based on IMD quintiles and education categories, separately. Multivariable models were adjusted for ethnicity, sex, smoking, age at inclusion, disease duration and body mass index (BMI) with a complete case approach. Goodness-of-fit was confirmed for all continuous variables (age at inclusion, disease duration and BMI) used in binary logistic regression using Hosmer-Lemeshow tests.

IMD distributions and educational attainment were compared between SjD and Sicca cohorts, and separately matched population controls. The whole West Midlands population (aged ≥16) was used to assess association between diagnosis (SjD or Sicca) and SES (IMD or educational attainment) with adjustment for sex, ethnicity, smoking status and age. The age variable was further condensed into four categories (16–34, 35–54, 55–74 and 75+) to increase case numbers for regression models.

#### Assessment of disease severity

Linear regression analysis was undertaken to assess for associations between IMD quintiles and select clinical markers as dependent variables. Education was not used as an independent variable due to the smaller dataset.

As IMD quintiles are an ordinal variable in its natural form, both continuous and categorical (using dummy variables) approaches were considered. The means, or the geometric means, of the variables with their CIs were assessed against the predicted mean from a linear regression model using continuous IMD variable. As predicted, values fell within the CIs, and linear models using a continuous IMD variable were considered a valid approach and used in both univariate and multivariable linear regressions. Models were adjusted for age at inclusion, ethnicity and smoking. Homoscedasticity of the models was assessed using scatter plots of regression standardised predicted values and regression standardised residuals.

Distribution of disease variables was visually assessed for normality. Distribution of Focus Score, ESSPRI, HADS anxiety and HADS depression was deemed normal; however, IgG, IgM, IgA, RF, Schirmer’s test, unstimulated salivary flow rates and ESSDAI had a skewed distribution and were therefore log_10_ transformed. Logged variables were individually checked against the continuous variable (age) to ensure good fit of the model.

#### Case-control matching

HSE 2019 cohort was used to identify matched controls with available IMD data for SjD and Sicca cohorts separately. Co-availability of education data was not a determinant for matching as this significantly reduced case numbers. One-to-one case control matching was undertaken using SPSS ‘FUZZY’ matching function with zero tolerances for matched variables. Matching variables were gender, ethnicity (dichotomous) and age (formatted into 10-year bands to match HSE format, that is, 25–34).

## Results

There were 256 SjD and 175 Sicca participants, and 972 suitable population controls based in West Midlands from the HSE 2019 cohort. Characteristics of the three cohorts are described in table 1. Further clinical characteristics of SjD and Sicca cohorts are described in table 2.

**Table 1:** Baseline characteristics table of HSE control, SjD and Sicca cohorts

		HSE n=972	SjD n=256	Sicca n=175	P values*		
		% (frequency/total) or median (IQR)			HSE vs SjD	SjD vs Sicca	HSE vs Sicca
Gender: female		51.9% (504)	94.1% (241)	89.7% (157)	**<0.001**	0.099	**<0.001**
Age (years)†	16–24	8.6% (67/782)	4.3% (11/255)	0.6% (1/173)	**0.009**	**0.025**	**<0.001**
	25–34	10.4% (81/782)	7.8% (20/255)	5.2% (9/173)			
	35–44	15.2% (119/782)	14.9% (38/255)	15.0% (26/173)			
	45–54	17.4% (136/782)	18.8% (48/255)	24.3% (42/173)			
	55–64	18.2% (142/782)	27.5% (70/255)	28.9% (50/173)			
	65–74	17.5% (137/782)	18.0% (46/255)	23.1% (40/173)			
	75+	12.8% (100/782)	8.6% (22/255)	2.9% (5/173)			
Ethnicity	White	76.7% (742/967)	65.0% (147/226)	87.8% (122/139)	**<0.001**	**<0.001**	**0.018**
	Asian	17.6% (172/967)	24.3% (55/226)	7.9% (11/139)			
	Black	3.0% (29/967)	4.4% (10/226)	3.6 (5/139)			
	Mixed	2.2% (21/967)	3.1% (7/226)	0.7% (1/139)			
	Other (inc. Arab)	0.3% (3/967)	3.1% (7/226)	—			
BMI (kg/m^2^)		n=972	n=256	n=175	0.823	**0.013**	**0.005**
		26.6 (22.3–30.7)	26.2 (23–30.1)	27.6 (23.8–32.4)			
IMD quintile	1 (most deprived)	33.4% (325/972)	34.6% (88/254)	20.8% (35/168)	0.272	**0.008**	**0.003**
	2	18.9% (184/972)	13.0% (33/254)	20.8% (35/168)			
	3	20.7% (201/972)	22.0% (56/254)	18.5% (31/168)			
	4	13.3% (129/972)	15.4% (39/254)	20.2% (34/168)			
	5 (least deprived)	13.7% (133/972)	15.0% (38/254)	19.6% (33/168)			
Educational attainment	No lower education	24.7% (189/765)	14.3% (22/154)	12.7% (15/118)	**<0.001**	**0.018**	**0.003**
	Lower secondary	25.0% (191/765)	27.3% (42/154)	19.5% (23/118)			
	Upper secondary	28.0% (214/765)	19.5% (30/154)	36.4% (43/118)			
	Degree or above	22.4% (171/765)	39.0% (60/154)	31.4% (37/118)			
Smoking: ever		51.0% (395/765)	29.2% (64/219)	47.3% (70/148)	**<0.001**	**<0.001**	0.413
Smoking status	Never	49.0% (380/765)	70.8% (155/219)	52.7% (78/148)	**<0.001**	**0.001**	0.516
	Current	15.6% (121/765)	5.9% (13/219)	12.2% (18/148)			
	Past	35.4% (274/765)	23.3% (51/219)	35.1% (52/148)			
Current antidepressant use: yes		11.6% (54/467)	17.3% (44/254)	19.1% (33/173)	**0.031**	0.877	**0.014**

HSE represents the West Midlands Health Survey England 2019 population cohort.

* Results for p values have been calculated using Mann-Whitney U test for continuous variables, and χ2 was used for all categorical variables, except for age and ethnicity for Sicca comparisons, where Fisher’s exact test was used. Bold p values are significant at p<0.05 for a two*-*sided significance*.*

† Data have been reported in categories as per HSE data format. SjD and Sicca cohorts only have participants aged 18 or older.

BMI, body mass index; HSE, Health Survey for England; IMD, indices of multiple deprivation; SjD, Sjögren’s disease.

**Table 2:** Clinical characteristics of SjD and Sicca cohorts

	SjD		Sicca		
	Median (IQR) or % (frequency/total)				P value*
Age at inclusion (years)					
SjD n=255	56.3 (43.9–65.3)		55.8 (46.4–64.3)		0.677
Sicca n=173* *					
Symptom duration (years)					
SjD n=248	5.1 (2.2–10.8)		4.7 (2.4–9.4)		0.325
Sicca n=163					
**Biological ** **variables**					
Positive anti-SSA/Ro antibody status	84% (215/256)		—		—
Rheumatoid factor level (U/mL)					
SjD n=234	32 (0–97.5)		0 (0)		**<0.001**
Sicca n=162* *					
Immunoglobulins (g/L)					
IgG level					
SjD n=246	15.93 (12.54–21.25)		10.68 (9.00–12.47)		**<0.001**
Sicca n=170* *					
IgA level					
SjD n=242	2.76 (1.99–3.53)		2.23 (1.62–3.02)		**<0.001**
Sicca n=170* *					
IgM level					
SjD n=246	1.13 (0.80–1.60)		0.98 (0.70–1.29)		**0.001**
Sicca n=169* *					
Focus score					
SjD n=113	1.5 (1.0–2.4)		0.29 (0–0.5)		**<0.001**
Sicca n=102* *					
Focus score≥1	77.0% (87/113)		4.9% (5/102)		**<0.001**
**Clinical variables* ***	**n **	**Median (IQR) **	**n **	**Median (IQR** ***)*** ** **	** **
Mean Schirmer test (mm)	241	4.50 (1.00–10.75)	166	10.25 (4.00–21.00)	**<0.001**

Unstimulated salivary flow (ml/min)		227	0.07 (0.02–0.15)	158	0.12 (0.04–0.26)	**<0.001 **
ESSDAI score		256	3.00 (1.00–6.75)	—	—	—
ESSPRI score		177	6.30 (4.70–7.70)	135	6.70 (4.70–8.00)	**0.045**
HAD scores	Anxiety	158	8.00 (5.00–12.00)	117	9.00 (6.00–12.00)	0.266
	Depression	159	6.00 (4.00–10.00)	118	7.00 (5.00–11.00)	0.147

Bold p values are significant at p<0.05 for a two-sided significance.

* Results for p values have been calculated using Mann-Whitney U-test for continuous variables and χ2 was used for all categorical variables, except ethnicity, where Fisher’s exact test was used.

ESSDAI, European Alliance of Associations for Rheumatology SjD disease activity index; ESSPRI, European Alliance of Associations for Rheumatology SjD patient-reported index; HAD, Hospital Anxiety and Depression; SjD, Sjögren’s disease.

SjD and Sicca cohorts were similar in sex, age (when compared as a continuous variable) and median disease duration. The HSE control cohort had a significantly higher proportion of male participants (p=<0.001) and notable differences in age distributions across categories.

Ethnicity of the participants differed significantly across all three groups, with the highest proportion of non-White participants in SjD (35%, p<0.001) and lowest in Sicca (12.2%, p≤0.001). Sicca participants also had a higher median BMI than SjD (p=0.013) and population controls (p=0.005).

Participants with SjD reported significantly lower rates of ever smoking (p<0.001) compared with other groups. Rates of ever smoking did not differ significantly between Sicca and controls.

Over 70% of SjD and 4.9% of Sicca participants had a focus score of one or above. The histological appearance in Sicca participants with a focus score ≥1 was not focal lymphocytic sialadenitis but instead was characterised by glandular atrophy and fibrosis.

IMD data were available for all participants residing at an English postcode; 254/256 (99.2%) SjD, 168/175 (96%) Sicca and 972/972 (100%) HSE control. Educational attainment data were available for 154/256 (60.2%) of SjD and 118/175 (67.4%) of Sicca participants, and 765/972 (78.7%) of controls. IMD quintile distributions and educational attainment differed significantly between SjD and Sicca cohorts, p=0.008 and p=0.018. Across both SES measures, participants with SjD had lower SES status compared with Sicca, as represented by a higher proportion without upper secondary education qualification and a higher proportion residing in 20% of most deprived areas of England (quintile 1). Additionally, fewer participants with SjDs resided in the least deprived areas (quintile 5). Interestingly, a higher proportion of participants with SjD had degree-level qualifications, suggesting a possible bimodal distribution.

IMD distributions of HSE suggest similar levels of deprivation as participants with SjD, thereby further highlighting Sicca as the less deprived. HSE controls had significantly lower levels of educational attainment than both disease cohorts (p<0.001 and p=0.003) with a larger proportion of individuals with no qualifications and a lower frequency of degree level qualifications.

### SES versus risk of disease

Tables 3 and 4 contain the separate binary regression models for association between risk of SjD (compared with Sicca) and IMD, and educational attainment. Both SES measures had a statistically significant association with risk of SjD, even after adjustment for confounders. In both models, non-white ethnicity was independently associated with increased odds of SjD.

**Table 3:** Multivariable logistic regression analysis of factors associated with risk of SjD vs Sicca—IMD based model

Independent variable		Univariate model			Multivariable model		
		N	OR (95% CI)	P value	N	OR (95% CI)	P value
IMD		422		**0.009**	276		**0.022**
	Quintile 1		Ref	—		Ref	——
	Quintile 2		0.38 (0.2 to 0.69)	**0.002**		0.26 (0.12 to 0.58)	**<0.001**
	Quintile 3		0.72 (0.4 to 1.29)	0.271		0.73 (0.32 to 1.39)	0.470
	Quintile 4		0.46 (0.25 to 0.84)	**0.011**		0.6 (0.25 to 1.39)	0.229
	Quintile 5		0.46 (0.25 to 0.84)	**0.012**		0.57 (0.24 to 1.33)	0.191
Ethnicity	(non-White)	365	3.86 (2.17 to 6.86)	**<0.001**		2.9 (1.33 to 6.34)	**0.008**
Smoking	(ever smoker)	367	0.46 (0.3 to 0.71)	**<0.001**		0.54 (0.3 to 0.96)	**0.037**
Gender	(male)	431	0.54 (0.27 to 1.11)	0.094		0.83 (0.29 to 2.41)	0.734
BMI (per kg/m^2^)		416	0.96 (0.93 to 0.99)	**0.006**		0.97 (0.93 to 1.01)	0.173
Disease duration (per year)		411	1.02 (1 to 1.05)	0.099		1.02 (0.99 to 1.06)	0.224
Age at inclusion (per decade)		428	0.94 (0.83 to 1.08)	0.423		0.98 (0.78 to 1.22)	0.844

Results are from binary logistic regression models. Initially, separate univariable models were produced for each factor, which included the stated number of cases. After excluding those with missing data for the factor, a multivariable model was then produced which included all factors; this used a complete-cases approach and hence was based on n=xx after exclusion of those with missing data. Bold p values are significant at p<0.05.

BMI, body mass index; IMD, indices of multiple deprivation; SjD, Sjögren’s disease.

**Table 4:** Multivariable logistic regression analysis of factors associated with risk of SjD vs Sicca—Education-based model

Independent variable		Univariate model			Multivariable model		
		N	OR (95% CI)	P value	N	OR (95% CI)	P value
Education		272		**0.020**	219		**0.026**
	No lower		Ref	——		Ref	—
	Lower secondary		1.25 (0.54 to 2.86)	0.605		1.48 (0.52 to 4.23)	0.467
	Upper secondary		0.48 (0.21 to 1.06)	0.070		0.41 (0.15 to 1.11)	0.078
	Degree or above		1.11 (0.51 to 2.4)	0.799		0.74 (0.27 to 2.01)	0.568
Ethnicity	(non-White)	365	3.86 (2.17 to 6.86)	**<0.001**		4.14 (1.65 to 10.39)	**0.003**
Smoking	(ever smoker)	367	0.46 (0.3 to 0.71)	**<0.001**		0.56 (0.3 to 1.04)	0.065
Gender	(male)	431	0.54 (0.27 to 1.11)	0.094		0.45 (0.14 to 1.48)	0.189
BMI (per kg/m^2^)		416	0.96 (0.93 to 0.99)	**0.006**		0.97 (0.92 to 1.02)	0.212
Disease duration (per year)		411	1.02 (1 to 1.05)	0.099		1.02 (0.98 to 1.07)	0.244
Age at inclusion (per decade)		428	0.94 (0.83 to 1.08)	0.423		1.01 (0.77 to 1.32)	0.971

Results are from binary logistic regression models. Initially, separate univariable models were produced for each factor, which included the stated number of cases. After excluding those with missing data for the factor, a multivariable model was then produced which included all factors; this used a complete-cases approach, hence was based on n=xx after exclusion of those with missing data. Bold p values are significant at p<0.05*.*

BMI, body mass index; SjD, Sjögren’s disease.

Due to missing values, binary logistic models were repeated with inclusion of a missing category to the smoking, education and ethnicity variables to assess for selection bias. The OR results were comparable; hence, formal models did not include a missing category (see online supplemental tables S1 and S2).

On univariate analysis for IMD (table 3), likelihood of SjD reduced across areas with less socioeconomic deprivation (as defined by higher IMD quintiles), p=0.009. Highest odds of SjD diagnosis were seen in most deprived areas (quintile 1), with odds of SjD reducing by 62% in quintile 2 (OR 0.38 (0.2, 0.69), p=0.002). After adjustment for confounders (n=276), there remained a significant reduction in odds of SjD in quintile two as opposed to the most deprived quintile, OR 0.26 (0.12, 0.58; p<0.001). Trends seen in the ORs for risk of disease across individual subcategories of education were not statistically significant (table 4).

Associations between SjD and individual IMD domains are presented in table 5. Across all domains, the general trend was of lower SjD risk with lower levels of deprivation. Income (p=0.014) and the supplementary IDAOPI domains (p=0.037) showed a statistically significant association with SjD.

**Table 5:** Univariate binary regression models for associations between IMD domains and diagnosis of SjD vs Sicca

	Quintile 1	Quintile 2	Quintile 3	Quintile 4	Quintile 5	Overall p value*
**Overall IMD**						**0.009**
N (%) of SjD	88/123 (71.5%)	33/68 (48.5%)	56/87 (64%)	39/73 (53.4%)	38/71 (53.5%)	
OR (95% CI)†	Ref	0.38 (0.2 to 0.69)	0.72 (0.4 to 1.29)	0.46 (0.25 to 0.84)	0.46 (0.25 to 0.84)	
P value†	—	**0.002**	0.271	**0.011**	**0.012**	
**Income domain**						**0.014**
N (%) of SjD	83/120 (69.2%)	45/74 (60.8%)	47/82 (57.3%)	36/70 (51.4%)	43/76 (56.6%)	
OR (95% CI)†	Ref	0.69 (0.38 to 1.27)	0.6 (0.33 to 1.07)	0.47 (0.26 to 0.87)	0.58 (0.32 to 1.06)	
P value†	—	0.234	0.085	**0.016**	0.074	
**Income deprivation affecting older people index (IDAOPI) domain**						**0.037**
N (%) of SjD	81/111 (73.0%)	45/81 (55.6%)	48/85 (56.6%)	38/67 (56.7%)	42/78 (53.8%)	
OR (95% CI)†	Ref	0.46 (0.25 to 0.85)	0.48 (0.26 to 0.88)	0.49 (0.26 to 0.92)	0.43 (0.23 to 0.80)	
P value†	—	**0.013**	**0.017**	**0.027**	**0.007**	
**Income deprivation affecting** **children index (IDACI) domain**						0.182
N (%) of SjD	75/113 (66.4%)	47/72 (65.3%)	45/73 (61.6%)	46/89 (51.7%)	41/75 (54.7%)	
OR (95% CI)†	Ref	0.95 (0.51 to 1.78)	0.81 (0.44 to 1.50)	0.54 (0.31 to 0.96)	0.61 (0.34 to 1.11)	
P value†	—	0.878	0.511	**0.035**	0.107	
**Employment domain**						0.177
N (%) of SjD	83/119 (69.7%)	36/65 (55.4%)	54/94 (57.4%)	43/76 (56.6%)	38/68 (55.9%)	
OR (95% CI)†	Ref	0.54 (0.29 to 1.01)	0.59 (0.33 to 1.03)	0.57 (0.31 to 1.03)	0.55 (0.3 to 1.02)	
P value†	—	0.053	0.064	0.062	0.058	
**Education, skills training domain**						0.106
N (%) of SjD	72/104 (69.2%)	49/78 (62.8%)	31/63 (49.2%)	42/76 (55.3%)	60/101 (59.4%)	
OR (95% CI)†	Ref	0.75 (0.40 to 1.4)	0.43 (0.23 to 0.82)	0.55 (0.3 to 1.02)	0.65 (0.37 to 1.16)	
P value†	—	0.365	**0.011**	0.056	0.143	
**Health deprivation and** **disability domain**						0.071
N (%) of SjD	71/104 (68.3%)	65/109 (59.6%)	56/86 (65.1%)	38/77 (49.4%)	24/46 (52.2%)	
OR (95% CI)†	Ref	0.69 (0.39 to 1.21)	0.87 (0.47 to 1.59)	0.45 (0.25 to 0.83)	0.51 (0.25 to 1.03)	
P value†	—	0.191	0.646	**0.011**	0.061	
**Crime domain**						0.399
N (%) of SjD	44/69 (63.8%)	72/112 (64.3%)	59/94 (62.8%)	42/80 (52.5%)	37/67 (55.2%)	
OR (95% CI)†	Ref	1.02 (0.55 to 1.91)	0.96 (0.50 to 1.83)	0.63 (0.33 to 1.21)	0.70 (0.35 to 1.39)	
P value†	—	0.944	0.900	0.166	0.311	
**Barriers to housing and services** **domain**						0.700
N (%) of SjD	74/114 (64.9%)	66/110 (60.0%)	52/86 (60.5%)	31/57 (54.4%)	31/55 (56.4%)	
OR (95% CI)†	Ref	0.81 (0.47 to 1.39)	0.83 (0.46 to 1.48)	0.64 (0.34 to 1.23)	0.70 (0.36 to 1.35)	
P value†	—	0.448	0.519	0.1847	0.284	
**Living environment domain**						0.759
N (%) of SjD	99/158 (62.7%)	51/84 (60.7%)	40/65 (61.5%)	39/67 (58.2%)	25/48 (52.1%)	
OR (95% CI)†	Ref	0.92 (0.54 to 1.59)	0.95 (0.53 to 1.73)	0.83 (0.46 to 1.49)	0.65 (0.34 to 1.24)	
P value†	—	0.767	0.875	0.531	0.192	

Data are reported as n/N (%) of patients with SjD for the national quintiles of each of the IMD domains, as well as ORs from univariable binary logistic regression models. Bold p values are significant at p<0.05*.*

* The overall p value, comparing across the five quintiles.

† ORs and p values are for comparisons between the stated quintile and quintile 1.

IMD, indices of multiple deprivation; SjD, Sjögren’s disease.

#### Population controls

**Figure 1:**
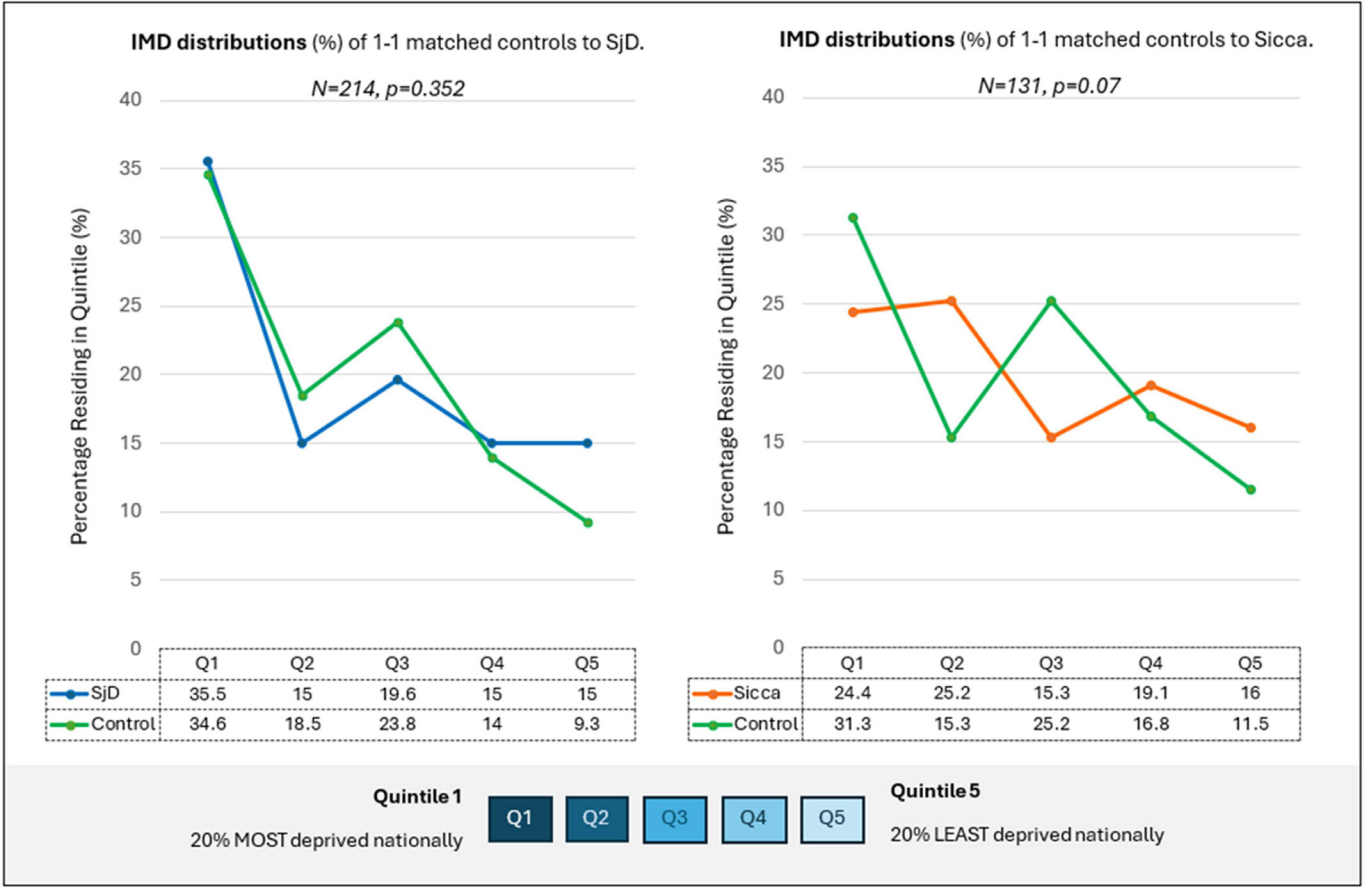
IMD distributions of one-to-one matched cohorts (SjD and separately Sicca) with population controls. Coloured lines represent percentage of participants (y-axis) in IMD quintiles (x-axis). Green is HSE population controls, blue is SjD and orange is Sicca. P values represent results of χ^2^ testing and are significant at p<0.05. HSE, health survey for England; IMD, indices of multiple deprivation; SjD, Sjögren’s disease*.*

IMD distributions of the matched cohorts can be seen in figure 1. Matched SjD (n=214) and population participants did not differ in IMD distribution (χ^2^; p=0.352). Comparison of matched Sicca (n=131) and population participants showed a trend towards association between lower levels of deprivation and Sicca, but this did not reach pre-defined statistical significance (p=0.07). When compared with the entire HSE population controls in logistic regression analysis, no association was found between IMD and risk of SjD (p=0.257). However, Sicca was associated with lower levels of deprivation (p=0.003). This remained true following adjustment for confounders with 264% increase in odds of Sicca in quintile two compared with quintile 1 (OR 2.64 (1.4, 4.97); p=0.003).

Baseline characteristics of matched cohorts and results of HSE regression models can be seen in online supplemental tables S3-S8.

Participants with SjD had significantly higher levels of educational attainment than their matched controls (p=0.010), with lower frequency of participants with no qualification (15.4% vs 23%) and higher frequency with

degree or above (39.2% vs 23%). A similar but not statistically significant trend was observed between Sicca and matched controls (p=0.096).

Regression models using entire HSE cohort showed that educational attainment, and in particular highest levels of attainment (degree or above) compared with having no qualifications, were significantly associated with increased odds of both SjD and Sicca (p<0.001 and p=0.003). Individuals with a degree or above had 359% and 246% higher odds of diagnosis of SjD and Sicca, respectively, than individuals with no qualifications (OR 3.585 (1.895, 6.782), p<0.001 and OR 2.46 (1.18, 5.11), p=0.016).

Non-white ethnicity was associated with increased odds of SjD across both SES models using general population controls (p=0.011 and p<0.001). However, only the education model showed association between non-white ethnicity and reduced odds of Sicca (p=0.003).

Due to concerns around bias in education models from inclusion of 16–17 year olds in HSE cohort, regression models were re-run without the 16–24 age category across all cohorts. Following exclusion, there were 244 SjD, 172 Sicca and 715 HSE participants. Multivariate education models showed comparable results whereby degree level qualification was significantly associated with increased risk of both SjD and Sicca (OR 3.40 (1.78,6.51), p <0.001 and OR 2.31 (1.11, 4.82), p=0.025).

#### SES versus disease features

SjD and Sicca groups had statistically significant differences across all clinical variables except for HAD anxiety and depression scores, see table 2.

When assessed for differences in clinical variables across levels of deprivation within participants with SjD, a trend of reducing titres/scores across successive quintiles was identified for IgG, IgA, unstimulated salivary flow and HAD depression scores (table 6).

**Table 6:** Distribution of median clinical variable scores across the IMD quintiles for patients with SjD

	Quintile 1	Quintile 2	Quintile 3	Quintile 4	Quintile 5	
	Median (IQR)					P value*
IgG level (g/L)	17.40 (13.71–23.34)	16.21 (13.64–23.70)	16.56 (12.10–22.44)	13.75 (10.26–16.80)	14.86 (11.63–19.61)	**<0.001**
IgA level (g/L)	2.80 (2.30–3.69)	3.08 (2.68–3.52)	2.61 (1.94–3.63)	2.35 (1.56–3.37)	2.16 (1.70–2.97)	**0.005**
IgM level (g/L)	1.17 (0.81–1.66)	1.10 (0.88–1.69)	1.00 (0.75–1.53)	1.23 (0.85–1.82)	1.20 (0.80–1.50)	0.835
Rheumatoid factor (U/mL)	33 (0–85)	22 (0–89)	52 (20–174)	0 (0–66)	28 (0–109)	0.996
Focus score	1.35 (0.80–2.46)	1.56 (1.29–2.29)	1.57 (1.14–2.56)	1.68 (1.24–2.16)	1.58 (0.89–2.20)	0.467
Unstimulated salivary flow (ml/min)	0.10 (0.04–0.19)	0.08 (0.01–0.23)	0.05 (0.01–0.13)	0.06 (0–0.10)	0.07 (0.02–0.12)	**0.006**
Mean Schirmer (mm)	6.0 (2.5–12.5)	5.5 (2.0–11.0)	2.0 (0.0–10.0)	3.5 (1.0–8.0)	4.5 (2.0–8.0)	0.077
ESSPRI	6.7 (5.0–7.7)	6.2 (5.3–7.7)	6.0 (4.5–7.0)	6.0 (3.9–8.0)	6.0 (3.7–7.3)	0.095
ESSDAI	3.0 (1.5–6.0)	3.0 (2.0–6.0)	3.0 (1.0–5.5)	5.0 (2.0–8.0)	2.5 (1.0–7.0)	0.851
HAD anxiety	9 (6–12)	11 (6–13)	7 (3–12)	9 (6–12)	7 (5–9)	0.291
HAD depression	7 (5–10)	9 (6–10)	6 (3–9)	7 (5–10)	5 (4–7)	**0.035**

* P values have been generated from Jonckheere-Terpstra test to determine statistical significance of the trend seen in clinical variables across IMD quintiles for patients with SjD. Bold p values are significant at p<0.05.

ESSDAI, European Alliance of Associations for Rheumatology SjD disease activity index; ESSPRI, European Alliance of Associations for Rheumatology SjD patient-reported index; HAD, hospital anxiety and depression; Ig, immunoglobulin; IMD, indices of multiple deprivation; SjD, Sjögren’s disease.

Similarly, linear regression models suggest that in SjD, IgG and IgA levels are negatively associated with lower levels of deprivation (ie, higher IMD quintiles; table 7). These associations remained significant following adjustment for age, smoking and ethnicity. Salivary flow and HAD depression score associations were not significant when confounders were considered. Interestingly, despite the differences in immunoglobulins, no associations were observed in focus scores across the quintiles.

**Table 7:** Associations between IMD quintile and disease markers in SjD

	N	Change per IMD quintile (95% CI)	P value*
IgG (% per IMD quintile)			
*Univariable*	244	−6.03% (−9.22% to −2.50%)	**<0.001**
*Multivariable*	184	−6.03% (−9.84% to −2.05%)	**0.003**
IgA (% per IMD quintile)			
*Univariable*	241	−6.24% (−10.05% to −2.28%)	**0.002**
*Multivariable*	182	−6.46% (−10.87% to −1.60%)	**0.010**
IgM (% per IMD quintile)			
*Univariable*	244	−0.74% (−5.35% to +4.10%)	0.759
*Multivariable*	184	−3.61% (−9.43% to +2.80%)	0.265
Rheumatoid factor (% per IMD quintile)			
*Univariable*	143	2.43% (−7.68% to 13.64%)	0.648
*Multivariable*	98	−3.38% (−16.07% to 11.24%)	0.629
Focus score (points per IMD quintile)			
*Univariable*	113	+0.01 (−0.11 to 0.13)	0.849
*Multivariable*	87	−0.04 (−0.19 to 0.12)	0.648
Unstimulated salivary rate (% per IMD quintile)			
*Univariable*	226	−16.06% (−24.13% to −7.13%)	**0.001**
*Multivariable*	167	−11.90% (−22.73% to +0.23%)	0.055
Mean Schirmer (% per IMD quintile)			
*Univariable*	239	−8.54% (−17.22% to 1.04%)	0.079
* Multivariable*	178	−2.05% (−13.50% to +10.66%)	0.732
ESSDAI (% per IMD quintile)			
*Univariable*	254	+1.07% (−6.34% to +9.07%)	0.782
*Multivariable*	190	+2.09% (−7.53% to +12.46%)	0.692
ESSPRI (points per IMD quintile)			
*Univariable*	176	−0.17 (−0.38 to +0.04)	0.114
*Multivariable*	151	−0.11 (−0.36 to +0.14)	0.388
HAD anxiety (points per IMD quintile)			
*Univariable*	157	−0.28 (−0.77 to 0.22)	0.270
*Multivariable*	131	0.13 (−0.44 to +0.70)	0.653
HAD depression (points per IMD quintile)			
*Univariable*	158	−0.46 (−0.87 to 0.04)	**0.033**
*Multivariable*	139	−0.12 (−0.62 to +0.38)	0.632

Bold p values are significant at p<0.05*.*

* Initially, univariable models were produced for each factor, with the IMD as a continuous covariable. Skewed variables were log_10_ transformed prior to analysis, with the resulting coefficients being anti-logged, and converted to percentage differences per IMD quintile. Variables that did not require log_10_ transformation are reported as points change per IMD quintile. Multivariable models were then produced, which additionally adjusted for age at inclusion, smoking status (never vs ever) and ethnicity (white vs non-white).

ESSDAI, European Alliance of Associations for Rheumatology SjD disease activity index; ESSPRI, European Alliance of Associations for Rheumatology SjD patient-reported index; HAD, hospital anxiety and depression; Ig, immunoglobulin; IMD, indices of multiple deprivation; SjD, Sjögren’s disease.

Among Sicca participants, a similar but smaller negative association between immunoglobulins and lower levels of deprivation was seen in univariate analysis. IgA had the highest percentage change per IMD quintile (−5.79% (−10.38%, −0.97%); p=0.019), followed by IgM (−5.44% (−10.34%, −0.27%); p=0.040) and IgG (−3.79% (6.31%, −1.20%); p=0.005). While the trend persisted following adjustment for confounders, the results did not reach statistical significance (p=0.064, p=0.183 and p=0.245 respectively). See online supplemental tables S10 and S11 for full results.

## Discussion

Our results demonstrate an association between SES, defined by two measures (IMD and educational attainment), and risk of SjD compared with Sicca controls. This association persisted following adjustment for covariates including ethnicity. Living in the 20% most deprived areas of England (ie, IMD quintile 1) compared with all other areas carries a 50% higher odds risk of SjD diagnosis compared with Sicca. Income-based domains appeared to be having the strongest contributions to the observed effects. It is uncertain why the largest observed impact appears to be across quintile 1 and quintile 2 rather than quintile 1 and 5. One possibility is that there are exposures/risk factors/behaviours which follow a U-shaped distribution and are most prevalent among the most deprived as well as the least. Therefore, the largest observed difference would be between the most deprived and the middle. A recognised example with such distribution is alcohol behaviour.^24^

Similar trends were seen with educational attainment, although statistical power was limited by the smaller sample size. However, our analysis against the general population suggests that having a degree level qualification is associated with an increased odds of any diagnosis—SjD or Sicca. It also revealed that it is the Sicca cohort that differs in their SES demographics, and that diagnosis of Sicca may be associated with lower levels of socioeconomic deprivation (as defined by IMD) and hence higher SES.

Despite the apparent lack of association between IMD and risk of SjD when compared with population controls, lower socioeconomic status was associated with higher immunoglobulins in SjD. This is of clinical importance as high immunoglobulins are considered a disease activity marker in SjD.^25^

While our results also demonstrate the effect of covariates such as sex, ethnicity, age and smoking on disease risk, these are recognised and reported in existing literature^10 26^ and were not the primary focus of this study.

Published literature focused on SES and risk of SjD is limited, conflicting and heterogenous in definition of both SjD and SES. Two large-scale studies of national electronic health records in the UK and Sweden^12 27^ assessed the risk of autoimmune diseases, including SjD, across different levels of area-based measures of deprivation (IMD and own neighbourhood deprivation index, respectively). Both found that the risk of autoimmune diseases was highest in most deprived areas, and a socioeconomic gradient was seen across most conditions, which reflects our findings. While both studies showed a trend towards increased risk of SjD in areas of higher deprivation, their results did not reach significance. However, a major limitation of both studies was the use of ICD10 classification codes for SjD that lack specificity, leading to probable inclusion of both SjD and Sicca. This recognised limitation led to a revision of code M35.0 in 2021 from ‘Sicca syndrome (Sjögren)’ to ‘Sjögren Syndrome’^28^ that postdated these studies. Overall accuracy of case definitions from ICD codes when compared with rheumatologist diagnosis has been found to vary between 68.9% and 82.9% for rheumatoid arthritis.^29^ Due to the high prevalence of sicca symptoms in the population, the heterogeneity of SjD and difficulties posed in accurate diagnosis, it is plausible that the accuracy of ICD codes for SjD will be less.

While education is a frequently used SES measure, factors such as ethnicity, immigration, birth cohorts and differences in educational systems affect its ability to reflect an individual’s SES.^13 30^ Three case-control studies reported no difference in education levels between SjD and control populations;^31^,^33^ one study^34^ and conference abstract^35^ reported an association between SjD and lower levels of education and one study found SjD patients to have under-representation of low as well as high educational attainment compared with population control.^2^ All studies had heterogenous definitions of SjD diagnosis, educational level and control populations and did not adjust for co-variates.

A small retrospective case-control study from 2005 in Sweden by Mostafavi *et al*^36^ found that manual versus non-manual paternal occupation at birth was associated with increased risk of SjD when compared with matched healthy controls. On the other hand, a matched case-control study by Olsson *et al*^37^ found no statistically significant difference in SjD risk between ‘blue-collar’ and ‘white-collar’ occupations of the participants themselves.

The mechanism behind the differential impact of SES on SjD and Sicca disease risk is uncertain. One possibility is that difference in health and healthcare seeking behaviours^38^ among SES groups are driving the difference in case diagnosis. Previous work by Alberts *et al*^39^ has shown that in some chronic conditions, higher educated individuals with an increased awareness of health (‘proto-professionalised individuals’) are more likely to receive specialist treatment. Hence, it may be that individuals of higher SES with sicca symptoms are better able to navigate the health system and obtain a Sicca diagnosis than symptomatic individuals of lower SES.^39^ This hypothesis may explain why high levels of educational attainment were associated with increased odds of any disease diagnosis (SjD or Sicca) when compared against population controls. This argument may also extend to IMD as a study by Skarda *et al*^40^ has previously found that those residing in the least deprived IMD quintile had the lowest risk of poor academic attainment. This potential bias does not apply to the comparison between SjD and Sicca as the referral pathways were the same for both.

SES may also be a consequence of disease rather than a cause. While this could theoretically contribute to IMD associations at presentation, educational attainment showed similar trends of association and education predated symptoms in the overwhelming majority of cases.

Alternatively, a biologically driven mechanism led by an environmental exposure is plausible and our findings of an association between IMD and immunoglobulins may support this. Although present in both SjD and Sicca cohorts, the relationship between levels of deprivation and IgG/IgA was more pronounced in SjD, with higher percentage change per IMD quintile. Unlike diagnosis, biological parameters such as immunoglobulins may be less influenced by health-seeking behaviours. As far as we are aware, there is no previous literature on the relationship between SES and biological markers of SjD disease activity.

Although data from rheumatoid arthritis studies are not directly translatable to SjD, an association between high socioeconomic deprivation and high disease activity (defined by composite disease activity scores)^15^ and autoantibody status^41^ has been repeatedly demonstrated. Similar associations have been reported for systemic lupus erythematosus, whereby SES has been correlated with disease activity levels, adverse long-term outcomes and autoantibody levels.^42 43^ Studies of the general population have also suggested that lower SES is associated with increased antibody levels to latent cytomegalovirus (CMV) and Herpes Simplex Virus 1 (HSV1) infections^44^ as well as with a more pro-inflammatory immune cell phenotype.^45^ The degree of immunomodulatory effects of deprivation and chronic stress also appears to vary based on age at exposure, with the largest impact in childhood years.^45^ Interestingly, both negative stressful life events and infections have been previously associated with increased risk of SjD.^3 4^

In the context of our findings that SES was associated with immunoglobulin levels and external evidence that SES may influence immune responses, we hypothesise that SES may modulate the clinical phenotype of salivary gland pathology in response to salivary gland insult. Those with higher SES would then present with a Sicca phenotype as opposed to a B cell hyperactive focal lymphocytic sialadenitis that characterises SjD. While non-SjD Sicca syndrome is a poorly characterised entity, it is not clinically insignificant as it has a similar symptom and quality of life burden to that seen in SjD.^46^ It is possible these sequelae are modulated by chronic stress, adverse event exposures or infections, all of which could be facilitated through SES of an individual, leading to immune system dysregulation.

The main limitations of our study are that it is a single-centre cohort and also incompleteness of education, ethnicity and smoking variables. This could introduce bias into our data, particularly as ethnicity has been shown to be related to SES. However, patterns of association are varied, and it is recognised that even within racial groups, SES appears to stratify health outcomes.^47^ We investigated the impact of the missing data with our supplementary analysis of the missing data category and this showed similar results. Additionally, while inclusion age of >16 in HSE cohort may impact on the validity of educational attainment data, models excluding the 16–24 groups showed very comparable results, making this unlikely.

As SES measures are contextual to the country and healthcare systems of the studied individuals, the generalisability of our findings may be limited to UK/English populations or populations with free healthcare systems. Additionally, as we used a composite area-based marker of deprivation (IMD), we cannot identify the driving factor for observed effect at the individual patient level. However, the results of our study support previously published work on SES in various populations and provide further hypotheses for exploration.

The use of a well-defined SjD cohort is a major strength of our study as well as the use of two comparator populations. Our work highlights the importance of control population selection in SjD research as differing conclusions would be reached if only using Sicca or population controls, as also evidenced by research on smoking risk.^10^

## Conclusions

Using a clearly defined SjD cohort and two SES measures, we have found that higher levels of socioeconomic deprivation are associated with increased risk of SjD compared with Sicca and, biologically, with higher immunoglobulin levels. The latter finding suggests an intriguing hypothesis that environmental factors may modulate the clinical phenotype of salivary gland pathology. The biological effects of SES in the context of SjD should be further explored.

## Supplementary material

10.1136/rmdopen-2025-006348 online supplemental file 1

## Data Availability

Data are available upon reasonable request.
