# Supplementary material for: Impact of socioeconomic deprivation on risk and disease activity of Sjögren’s disease
Source: RMD Open. 2026 Mar 4;12(1):e006348. doi: 10.1136/rmdopen-2025-006348 (PMC12970116; doi:10.1136/rmdopen-2025-006348)
Supplement: online supplemental file 1 [file rmdopen-12-1-s001.pdf]

Supplementary Materials

Impact of socioeconomic deprivation on risk and disease activity of Sjogren's Disease.

Aliaksandra Baranskaya[1,2,3], Matilde Bandeira[1,3,4,5], Abdullah Nadeem[6], Simon J. Bowman[1,2,3], Valentina Pucino[1,3] and Benjamin A. Fisher[1,2,3]

1 School of Infection, Inflammation and Immunology, University of Birmingham, Birmingham, UK

2 NIHR Birmingham Biomedical Research Centre, University Hospitals Birmingham, Birmingham, UK.

3 Queen Elizabeth Hospital, University Hospitals Birmingham NHS Foundation Trust, Department of Rheumatology, Birmingham, United Kingdom

4 Rheumatology Department, Unidade Local de Saúde Santa Maria, Centro Académico de Medicina de Lisboa (CAML), Lisboa, Portugal

5 Faculdade de Medicina, Universidade de Lisboa, CAML, Lisboa, Portugal

6 University of Leicester Medical School, University of Leicester, Leicester, UK

Corresponding author:

Professor Benjamin A Fisher – b.fisher@bham.ac.uk

Department of Inflammation and Ageing
School of Infection, Inflammation and Immunology,
College of Medicine and Health
University of Birmingham
Edgbaston
Birmingham
B15 2TT

Supplementary Methods and Tables:

The following variables from the HSE survey were used:

- Government Office Region (*GOR1*) variable used to identify participants based in West Midlands.
- Gender (*Sex*), BMI (*BMIVal*)
- Ethnicity (*origin2*) was reformatted to dichotomous White/Non-White variable.
- Age (*ag16g10*)– in HSE age is presented as bands starting from 16 for adults, we used the 10-year band variable (i.e 16-24). We reformatted OASIS data to match this for analysis. Only participants >16 were used in analysis as it was not possible to filter HSE data for >18 only.
- Several smoking variables are available, we used *cigsta3_19* variable (Cigarette Smoking Status: Current/Ex-Reg/Never-Reg) as this was most reflective of the smoking variable used in OASIS. This was reformatted into Ever/Never smoking variable.
- IMD quintiles (*qimd19*) - the *qimd19* variable was transformed to reflect the format of our IMD variable whereby quintile 1 represents 20% most deprived, and quintile 5 is 20% least deprived.
- Educational Attainment (*topqual3*) - the *topqual3* variable was transformed to match the format with OASIS as described in methods.

Table S1 – Multivariable analysis of factors associated with SjD, IMD-based models.

		**Univariate Model**			**Univariate Model with missing category**			**Multivariable Model**			**Multivariable Model with missing category**		
Independent variable		*N*	OR [95% C.I.]	P-value	*N*	OR [95% C.I.]	P-value	*N*	OR [95% C.I.]	P-value	*N*	OR [95% C.I.]	P-value
**IMD Quintile**		*422*		**0.009**	*422*		**0.009**	*276*		**0.022**	*386*		**0.040**
	Quintile 1		Ref	**-**		Ref	**-**		Ref	**-**		Ref	**-**
	Quintile 2		0.38 [0.2, 0.69]	**0.002**		0.38 [0.2, 0.69]	**0.002**		0.26 [0.12, 0.58]	**<0.001**		0.35 [0.17, 0.7]	**0.003**
	Quintile 3		0.72 [0.4, 1.29]	0.271		0.72 [0.4, 1.29]	0.271		0.73 [0.32, 1.39]	0.470		0.71 [0.36, 1.43]	0.34
	Quintile 4		0.46 [0.25, 0.84]	**0.011**		0.46 [0.25, 0.84]	**0.011**		0.6 [0.25, 1.39]	0.229		0.52 [0.26, 1.06]	0.072
	Quintile 5		0.46 [0.25, 0.84]	**0.012**		0.46 [0.25, 0.84]	**0.012**		0.57 [0.24, 1.33]	0.191		0.48 [0.23, 0.98]	**0.044**
**Ethnicity**		*365*			*431*		**<0.001**						**0.002**
	White		Ref	**-**		Ref	**-**		Ref	**-**		Ref	**-**
	Non-White		3.86 [2.17, 6.86]	**<0.001**		3.86 [2.17, 6.86]	**<0.001**		2.9 [1.33, 6.34]	**0.008**		2.98 [1.52, 5.84]	**0.001**
	Missing					0.69 [0.40, 1.19]	0.181					0.71 [0.38, 1.33]	0.279
**Smoking**		*367*			*431*		**0.002**						**0.043**
	Never smoker		Ref	**-**		Ref	**-**		Ref	-		Ref	-
	Ever smoker		0.46 [0.3, 0.71]	**<0.001**		0.46 [0.3, 0.71]	**<0.001**		0.54 [0.3, 0.96]	**0.037**		0.53 [0.31, 0.89]	**0.017**
	Missing					0.69 [0.39, 1.21]	0.198					0.61 [0.32, 1.16]	0.130
**Gender**	(Male)	*431*	0.54 [0.27, 1.11]	0.094	*431*	0.54 [0.27, 1.11]	0.094		0.83 [0.29, 2.41]	0.734		0.7 [0.3, 1.61]	0.402
**BMI (per kg/m2)**		*416*	0.96 [0.93, 0.99]	**0.006**	*416*	0.96 [0.93, 0.99]	**0.006**		0.97 [0.93, 1.01]	0.173		0.97 [0.93, 1]	0.069
**Disease duration (per year)**		*411*	1.02 [1, 1.05]	0.099	*411*	1.02 [1, 1.05]	0.099		1.02 [0.99, 1.06]	0.224		1.03 [1, 1.07]	**0.034**
**Age at inclusion (per decade)**		*428*	0.94 [0.83, 1.08]	0.423	*428*	0.94 [0.83, 1.08]	0.423		0.98 [0.78, 1.22]	0.844		1.01 [0.85, 1.21]	0.898

Results are from binary logistic regression models. Initially, separate univariable models were produced for each factor, which included the stated number of cases, after excluding those with missing data for the factor. A multivariable model was then produced which included all factors; this used a complete-cases approach, hence was based on N=xx after exclusion of those with missing data. Bold p-values are significant at p<0.05.

Table S2 – Multivariable analysis of factors associated with SjD, education-based model

		**Univariate Model**			**Univariate with missing category**			**Multivariable Model**			**Multivariable with missing category**		
Independent variable		*N*	OR [95% C.I.]	P-value	*N*	OR [95% C.I.]	P-value	*N*	OR [95% C.I.]	P-value	*N*	OR [95% C.I.]	P-value
**Education**		*272*		**0.020**	*431*		***0.016***	*129*		***0.026***	*395*		**0.003**
	No lower		Ref	**-**		Ref	-		Ref	**-**		Ref	**-**
	Lower secondary		1.25 [0.54, 2.86]	0.605		1.25 [0.54, 2.86]	0.605		1.48 [0.52, 4.23]	0.467		1.38 [0.53, 3.57]	0.507
	Secondary		0.48 [0.21, 1.06]	0.070		0.48 [0.21, 1.06]	0.070		0.41 [0.15, 1.11]	0.078		0.41 [0.16, 1.02]	0.055
	Degree or above		1.11 [0.51, 2.4]	0.799		1.11 [0.51, 2.4]	0.799		0.74 [0.27, 2.01]	0.568		1.02 [0.41, 2.54]	0.972
	Missing					1.22 [0.59. 2.54]	0.594					1.63 [0.64, 4.20]	0.308
**Ethnicity**		*365*			*431*		**<0.001**						**<0.001**
	White		Ref	**-**		Ref	**-**		Ref	**-**		Ref	**-**
	Non-White		3.86 [2.17, 6.86]	**<0.001**		3.86 [2.17, 6.86]	**<0.001**		4.14 [1.65, 0.39]	**0.003**		3.8 [1.98, 7.32]	**<0.001**
	Missing					0.69 [0.40, 1.19]	0.181					0.69 [0.37, 1.29]	0.245
**Smoking**		*367*			*431*		**0.002**						**0.023**
	Never smoker		Ref	**-**		Ref	**-**		Ref	-		Ref	-
	Ever smoker		0.46 [0.3, 0.71]	**<0.001**		0.46 [0.3, 0.71]	**<0.001**		0.56 [0.3, 1.04]	0.065		0.59 [0.35, 0.98]	**0.040**
	Missing					0.69 [0.39, 1.21]	0.198					0.41 [0.19, 0.89]	**0.024**
**Gender**	(Male)	*431*	0.54 [0.27, 1.11]	0.094	*431*	0.54 [0.27, 1.11]	0.094		0.45 [0.14, 1.48]	0.189		0.53 [0.24. 1.18]	0.118
**BMI (per kg/m2)**		*416*	0.96 [0.93, 0.99]	**0.006**	*416*	0.96 [0.93, 0.99]	**0.006**		0.97 [0.92, 1.02]	0.212		0.97 [0.93, 1]	0.073
**Disease duration (per year)**		*411*	1.02 [1, 1.05]	0.099	*411*	1.02 [1, 1.05]	0.099		1.02 [0.98, 1.07]	0.244		1.03 [1, 1.06]	**0.047**
**Age at inclusion (per decade)**		*428*	0.94 [0.83, 1.08]	0.423	*428*	0.94 [0.83, 1.08]	0.423		1.01 [0.77, 1.32]	0.971		1.07 [0.9, 1.28]	0.434

Results are from binary logistic regression models. Initially, separate univariable models were produced for each factor, which included the stated number of cases, after excluding those with missing data for the factor. A multivariable model was then produced which included all factors; this used a complete-cases approach, hence was based on N=xx after exclusion of those with missing data. Bold p-values are significant at p<0.05.

Table S3 - Baseline characteristics of 1-1 matched populations controls and SjD (those with available IMD data were matched for sex, age and ethnicity).

		**Control (N=214)**	**SjD (N=214)**	**p- Value**
		N (%)	N (%)	
Gender (Female)		201 (93.9%)	201 (93.9%)	1.000
Ethnicity (White)		144 (67.3%)	144 (67.3%)	1.000
Age bands (years)	16-34	27 (12.6%)	27 (12.6%)	1.000
	35-54	76 (35.5%)	76 (35.5%)	
	55-74	91 (42.5%)	91 (42.5%)	
	75+	20 (9.3%)	20 (9.3%)	
IMD	Quintile 1 (Most deprived)	74 (34.6%)	76 (35.5%)	0.352
	Quintile 2	39 (18.2%)	32 (15.0%)	
	Quintile 3	51 (23.8%)	42 (19.6%)	
	Quintile 4	30 (14.0%)	32 (15.0%)	
	Quintile 5 (Least deprived)	20 (9.3%)	32 (15.0%)	
Educational attainment		*N=209*	N=130	**0.010**
	No lower education	48 (23.0%)	20 (15.4%)	
	Lower secondary	56 (26.8%)	33 (25.4%)	
	Higher secondary	57 (27.3%)	26 (20.0%)	
	Degree or above	48 (23.0%)	51 (39.2%)	
Smoking (Ever Regular)*		*N=214*	*N=181*	0.721
		65 (30.4%)	58 (32.0%)	

* *cigsta3_19* variable used.
P-Values are generated from Chi^2 tests. Bold p-values are significant at $p<0.05$.

Table S4 - Baseline characteristics of 1-1 matched populations controls and Sicca (those with available IMD data were matched for sex, age and ethnicity).

		Control (N=131)	Sicca (N=131)	**p-Value**
		N (%)	N (%)	
Gender (Female)		118 (90.1%)	118 (90.1%)	1.000
Ethnicity (White)		114 (87.0%)	114 (87.0%)	1.000
Age bands (years)	16-34	9 (6.9%)	9 (6.9%)	1.000
	35-54	50 (38.2%)	50 (38.2%)	
	55-74	68 (51.9%)	68 (51.9%)	
	75+	4 (3.1%)	4 (3.1%)	
IMD	Quintile 1 (Most deprived)	41 (31.3%)	32 (24.4%)	0.070
	Quintile 2	20 (15.3%)	33 (25.2%)	
	Quintile 3	33 (25.2%)	20 (15.3%)	
	Quintile 4	22 (16.8%)	25 (19.1%)	
	Quintile 5 (Least deprived)	15 (11.5%)	21 (16.0%)	
Educational attainment		*N=127*	*N=92*	0.096
	No lower education	28 (22.0%)	14 (15.2%)	
	Lower secondary	35 (27.6%)	16 (17.4%)	
	Higher secondary	34 (26.8%)	33 (35.9%)	
	Degree or above	30 (23.6%)	29 (31.5%)	
Smoking (Ever Regular)*		*N=131*	*N=109*	0.083
		48 (36.6%)	52 (47.7%)	

* *cigsta3_19* variable used.
P-Values are generated from Chi^2 tests. Bold p-values are significant at $p<0.05$.

	Univariate			Multivariable		
Independent variable	*N*	OR (95% C.I.)	p-Value	*N*	OR (95% C.I.)	p-Value
**IMD**	*1226*		0.278	*964*		0.257
Quintile 1		Ref	-		Ref	-
Quintile 2		0.662 (0.427-1.207)	0.066		0.659 (0.380, 1.145)	0.139
Quintile 3		1.029 (0.705, 1.502)	0.882		0.839 (0.509, 1.383)	0.491
Quintile 4		1.117 (0.727, 1.714)	0.614		1.182 (0.680, 2.053)	0.553
Quintile 5		1.055 (0.686, 1.623)	0.807		1.257 (0.717, 2.204)	0.424
**Gender (male)**	*1228*	0.067 (0.039, 0.115)	**<0.001**		0.062 (0.032, 0.120)	**<0.001**
**Ethnicity (non-white)**	*1193*	1.772 (1.298, 2.420)	**<0.001**		2.789 (1.772, 4.388)	**<0.001**
**Age**	*1037*		**0.005**			**0.003**
16-34		Ref	**-**		Ref	**-**
35-54		1.610 (1.019, 2.545)	**0.041**		1.636 (0.916, 2.922)	0.096
55-74		1.985 (1.274, 3.093)	**0.002**		2.762 (1.557, 4.902)	**<0.001**
75+		1.050 (0.575, 1.918)	0.873		1.847 (0.872, 3.909)	0.109
**Smoking (ever reg)***	*994*	0.698 (0.504, 0.967)	**0.031**		1.223 (0.819, 1.828)	0.325

* *cigsta3_19* variable used.

Results are from binary logistic regression models. Initially, separate univariable models were produced for each factor, which included the stated number of cases, after excluding those with missing data for the factor. A multivariable model was then produced which included all factors; this used a complete-cases approach, hence was based on N=xx after exclusion of those with missing data. Bold p-values are significant at p<0.05.

Table S5. Binary regression models for risk of SjD against HSE control cohort – IMD model.

Table S6 - Binary regression models for risk of Sicca against HSE control cohort – IMD model.

	Univariate			Multivariable		
Independent variable	*N*	OR (95% C.I.)	p-Value	*N*	OR (95% C.I.)	p-Value
**IMD**	*1140*		**0.003**	*883*		**0.016**
Quintile 1		Ref	**-**		Ref	**-**
Quintile 2		1.766 (1.069, 2.919)	**0.026**		2.640 (1.402, 4.972)	**0.003**
Quintile 3		1.432 (0.856, 2.395)	0.171		1.056 (0.523, 2.132)	0.880
Quintile 4		2.447 (1.464, 4.093)	**<0.001**		1.824 (0.909, 3.660)	0.091
Quintile 5		2.304 (1.374, 3.862)	**0.002**		2.029 (0.992, 4.153)	0.053
**Gender (male)**	*1147*	0.123 (0.075, 0.204)	**<0.001**		0.091 (0.045, 0.185)	**<0.001**
**Ethnicity (non-white)**	*1106*	0.460 (0.271, 0.780)	**0.004**		0.824 (0.410, 1.654)	0.586
**Age**	*955*		**<0.001**			**<0.001**
16-34		Ref	**-**		Ref	**-**
35-54		3.947 (1.972, 7.900)	**<0.001**		3.430 (1.380, 8.525)	**0.008**
55-74		4.774 (2.411, 9.452)	**<0.001**		5.374 (2.213, 13.047)	**<0.001**
75+		0.740 (0.246, 2.230)	0.593		0.740 (0.176, 3.117)	0.681
**Smoking (ever reg)***	*923*	1.518 (1.085, 2.162)	**0.021**		2.097 (1.318, 3.339)	**0.002**

* *cigsta3_19* variable used.

Results are from binary logistic regression models. Initially, separate univariable models were produced for each factor, which included the stated number of cases, after excluding those with missing data for the factor. A multivariable model was then produced which included all factors; this used a complete-cases approach, hence was based on N=xx after exclusion of those with missing data. Bold p-values are significant at p<0.05.

Table S7 - Binary regression models for risk of **SjD** against HSE control cohort – **Education Model**.

	Univariate			Multivariable		
Independent variable	*N*	OR (95% C.I.)	p-Value	*N*	OR (95% C.I.)	p-Value
**Education**	*919*		**<0.001**	*897*		**<0.001**
no lower education		Ref	-		**Ref**	-
lower secondary		1.889 (1.086, 3.286)	**0.024**		1.799 (0.953, 3.398)	0.070
secondary		1.204 (0.672, 2.159)	0.533		1.456 (0.761, 2.788)	0.257
Degree or above		3.014 (1.773, 5.124)	**<0.001**		3.585 (1.895, 6.782)	**<0.001**
**Gender (male)**	*1228*	0.067 (0.039, 0.115)	**<0.001**		0.051 (0.022, 0.118)	**<0.001**
**Ethnicity (non-white)**	*1193*	1.772 (1.298, 2.420)	**<0.001**		1.907 (1.158, 3.139)	**0.011**
**Age**	*1037*		**0.005**			**<0.001**
16-34		Ref	**-**		Ref	**-**
35-54		1.610 (1.019, 2.545)	**0.041**		2.126 (1.028, 4.397)	**0.042**
55-74		1.985 (1.274, 3.093)	**0.002**		4.300 (2.060, 8.975)	**<0.001**
75+		1.050 (0.575, 1.918)	0.873		4.325 (1.725, 10.845)	**0.002**
**Smoking (ever reg)***	*994*	0.698 (0.504, 0.967)	**0.031**		0.676 (0.439, 1.043)	0.077

* *cigsta3_19* variable used.
Results are from binary logistic regression models. Initially, separate univariable models were produced for each factor, which included the stated number of cases, after excluding those with missing data for the factor. A multivariable model was then produced which included all factors; this used a complete-cases approach, hence was based on N=xx after exclusion of those with missing data. Bold p-values are significant at p<0.05.

Table S8 - Binary regression models for risk of **Sicca** against HSE control cohort - **Education** model.

	Univariate			Multivariable		
Independent variable	*N*	OR (95% C.I.)	p-Value	*N*	OR (95% C.I.)	p-Value
**Education**	*883*		**0.004**	*857*		**0.003**
no lower education		Ref	**-**		Ref	**-**
lower secondary		1.517 (0.768, 2.997)	0.230		0.785 (0.356, 1.733)	0.550
secondary		2.532 (1.363, 4.704)	**0.003**		1.905 (0.945, 3.840)	0.072
Degree or above		2.726 (1.445, 5.143)	**0.002**		2.459 (1.183, 5.108)	**0.016**
**Gender (male)**	*1147*	0.123 (0.075, 0.204)	**<0.001**		0.107 (0.052, 0.218)	**<0.001**
**Ethnicity (non-white)**	*1106*	0.460 (0.271, 0.780)	**0.004**		0.036 (0.439, 0.948)	**0.036**
**Age**	*955*		**<0.001**			**<0.001**
16-34		Ref	**-**		Ref	**-**
35-54		3.947 (1.972, 7.900)	**<0.001**		3.215 (1.284, 8.053)	**0.013**
55-74		4.774 (2.411, 9.452)	**<0.001**		5.644 (2.299, 13.851)	**<0.001**
75+		0.740 (0.246, 2.230)	0.593		1.017 (0.651, 1.646)	0.681
**Smoking (ever reg)***	*923*	1.518 (1.085, 2.162)	**0.021**		1.035 (0.651, 1.646)	0.883

* *cigsta3_19* variable used.
Results are from binary logistic regression models. Initially, separate univariable models were produced for each factor, which included the stated number of cases, after excluding those with missing data for the factor.

A multivariable model was then produced which included all factors; this used a complete-cases approach, hence was based on N=xx after exclusion of those with missing data. Bold p-values are significant at p<0.05.

		Quintile 1	Quintile 2	Quintile 3	Quintile 4	Quintile 5	
	**Total N**	Median (IQR)					**p-Value***
**IgG level (g/L)**	163	11.66 (10.48-14.54)	11.16 (9.30-12.46)	10.44 (8.69-11.70)	10.31 (8.90-12.21)	9.78 (8.15-11.62)	**0.002**
**IgA level (g/L)**	163	2.79 (1.94-3.51)	2.34 (1.69-3.27)	1.90 (1.52-2.39)	2.05 (1.47-2.89)	2.17 (1.51-3.01)	**0.019**
**IgM level (g/L)**	162	1.00 (0.78-1.58)	1.02 (0.65-1.27)	1.05 (0.80-1.31)	0.87 (0.73-1.28)	0.84 (0.65-1.15)	0.082
**Rheumatoid factor (U/mL)**	155	0 (0)	0 (0)	0 (0)	0 (0)	0 (0)	0.131
**Unstimulated salivary flow (ml/min)**	151	0.10 (0.04-0.28)	0.07 (0.04-0.39)	0.14 (0.02-0.22)	0.14 (0.06-0.28)	0.12 (0.03-0.20)	0.624
**Mean Schirmer (mm)**	159	8.50 (4.50-24)	15.5 (3.50-20)	15.0 (6-30.5)	9.5 (2.5-18.5)	7.5 (4-17.5)	0.629
**ESSPRI**	130	6.85 (4.50-8.30)	6.30 (5.3-8)	6.7 (5-8.3)	7.3 (4.5-7.7)	6.85 (4-7.7)	0.649
**HAD Anxiety**	112	12 (7-15)	10 (6-12)	9 (6-12)	10 (6-12)	8 (2-12)	0.195
**HAD Depression**	113	9 (6-11)	7 (4-10)	7 (3-10)	8 (6-11)	7 (2-11)	0.689

*P-values have been generated from Jonckheere-Terpstra test to determine statistical significance of the trend seen in clinical variables across IMD quintiles for patients with Sicca. Bold p-values are significant at p<0.05.

Table S9 - Distribution of median clinical variable scores across the IMD quintiles for patients with Sicca.

Table S10 - Associations between IMD quintiles and disease markers in Sicca.

	N	Change per IMD Quintile (95% CI)	p-Value *
**IgG (% per IMD Quintile)**			
Univariable	*163*	-3.79% (-6.31%, -1.20%)	**0.005**
Multivariable	*108*	-1.96% (-5.21%, 1.39%)	0.245
**IgA (% per IMD Quintile)**			
Univariable	*163*	-5.79% (-10.38%, -0.97%)	**0.019**
Multivariable	*107*	-5.53% (-11.06%, 0.34%)	0.064
**IgM (% per IMD Quintile)**			
Univariable	*162*	-5.44% (-10.34%, -0.27%)	**0.040**
Multivariable	*106*	-4.93% (-11.78%, +2.45%)	0.183
**Unstimulated salivary rate (% per IMD Quintile)**			
Univariable	*141*	-3.38% (-16.09%, -11.26%)	0.630
Multivariable	*90*	0.33% (-15.55%, +19.20%)	0.970
**Mean Schirmer (% per IMD Quintile)**			
Univariable	*149*	-0.25% (-11.80%, +12.80%)	0.968
Multivariable	*96*	5.19% (-11.36%, +24.82%)	0.559
**ESSPRI (points per IMD Quintile)**			
Univariable	*130*	-0.06 (-0.33, +0.21)	0.645
Multivariable	*100*	-0.035 (-0.36, +0.29)	0.832
**HAD Anxiety (points per IMD Quintile)**			
Univariable	*112*	-0.42 (-1.09, 0.26)	0.222
Multivariable	*91*	-0.33 (-1.10, +0.44)	0.391
**HAD Depression (points per IMD Quintile)**			
Univariable	*113*	-0.18 (-0.75, -0.39)	0.534
Multivariable	*92*	0.08 (-0.56, +0.73)	0.804

**Initially, univariable models were produced for each factor, with the IMD as a continuous covariable. Skewed variables were log_{10} transformed prior to analysis, with the resulting coefficients being anti-logged, and converted to percentage differences per IMD quintile. Variables that did not require log10*

transformation are reported as points changer per IMD quintile. Bold p-values are significant at p<0.05.

Rheumatoid factor was not assessed in linear models for Sicca cohort as due to large number of null values, the case number of the logged variable was too small for meaningful assessment.
